# Thalassemia care in Nepal: In dire need of improvement

**DOI:** 10.1002/jha2.681

**Published:** 2023-03-20

**Authors:** Bishesh Sharma Poudyal, Anirudra Devkota, Peter Kouides

**Affiliations:** ^1^ Clinical Hematology and Bone Marrow Transplant Unit Civil Service Hospital Kathmandu Nepal; ^2^ Gandaki Medical College Pokhara Nepal; ^3^ Mary M. Gooley Hemophilia Center Rochester New York USA

1

In the developed world, thalassemia has become a disorder like any other chronic disease that requires regular follow‐up [1]. Individuals with mild variants of the condition can have normal or almost normal lives, but those with severe types will need ongoing monitoring and treatment, including regular blood transfusions, iron chelation, or allogeneic stem cell transplantation. As of today, allogeneic stem cell transplantation is the only curative modality for thalassemia major. Gene therapy remains another hope [2], as well as novel agents like luspatercept [3]. Sickle cell disease and thalassemia are the most common hemoglobinopathies detected in Nepal. Sickle cell is more prevalent in the western region of Nepal, particularly among Tharu ethnic groups. In contrast, the prevalence of thalassemia is widespread across the nation and all ethnic groups [4]. Thalassemia is associated with significant consumption of healthcare resources [5]. In Nepal, the financial burdens associated with thalassemia diagnosis and treatment have not been studied. We carried out this study to document the social–economic determinants, treatment challenges, and complications associated with β‐thalassemia major in Nepalese thalassemia patients registered with the Nepal Thalassemia Society.

Medical charts of 54 thalassemia major patients registered at the Civil Service Hospital were retrospectively analyzed to document demographics, height, weight, age of diagnosis, the total number of transfusions, transfusion frequency, and serum ferritin. Fifteen questionnaires were prepared, and each patient or patient relatives were interviewed, focusing mainly on transfusion adherence and adherence to iron chelation therapy. Tanner scoring to document sexual development was done on all patients on the same day of the interview. The mean, median, standard deviation, ranges and frequency, and percentage were calculated for the variables.

The median age of the patients was 17 years (3–34 years), and the median age of diagnosis was 18 months (range 4–192 months). Overall, 57% (31) of patients were male, and 43% (23) were female, in which 71% (38) of patients failed to maintain hemoglobin above 9.5 gm% (the recommended level) primarily because of the unavailability of red blood cells. Table 1 represents the demographics and laboratory characteristics of 54 thalassemia major patients.

**TABLE 1:** Demographics and laboratory characteristics of 54 thalassemia major patients

Total β‐thalassemia major patients	54
Median age of patients	17 years
Median age at diagnosis	18 months
Males (%)	57
Females (%)	43
Mean serum ferritin	4486.12 ng/mL

The mean serum ferritin was 4486.12 ng/mL (range 469–9585 ng/mL). Overall, 80% (43) of patients reported interrupted use of iron‐chelating agents because of frequent shortages of medicines, and 54.1% (29) of females of reproductive age had primary amenorrhea. Overall, 71.15% (38) of the patients were sexually underdeveloped, and 93% (50) of patients were found to have stunted growth. Overall, 7.6% (4) had cardiac complications, with mitral and tricuspid regurgitation being the most common, and 5.5% (3) had hypothyroidism. The average cost of thalassemia care was 70,000 NRS (∼533 USD) in pediatric patients and 250,000 NRS (∼1906 USD) in adult patients per annum. This includes the cost of blood transfusion, commute to the transfusion center, and iron‐chelating agents. Table 2 represents complications of the disease and treatment.

**TABLE 2:** Complications of the disease and treatment

Iron overload (%)	96.2
Stunted growth (%)	93
Sexually underdeveloped (%)	71.15
Primary amenorrhea (%)	54.1
Cardiac complications (%)	7.6

To the best of our knowledge, this is the first study that sheds light on the reality of β‐thalassemia major patients in Nepal. We observed that 72% (39) of patients had to interrupt regular blood transfusion due to the unavailability of packed red blood cells, let alone in the country's capital city, not even considering patients outside Kathmandu valley where intuitively unavailability would be higher.

Iron‐cheating agents are known to improve the survival of thalassemia major patients and are therefore considered the backbone of thalassemia care [6]. World Health Organization has listed iron‐chelating agents in an essential medicine category [7]. The unavailability or frequent shortages of iron‐chelating agents that are enlisted as an essential medicine is a significant concern. Furthermore, the finding of underdevelopment of sexual characteristics in 71.15% of patients secondary to inadequate iron chelation is very alarming. The right to health has been guaranteed in the constitution of Nepal. Therefore, we believe that it is the sole duty of the government to make these medicines available at any cost to improve thalassemia care in the country.

Bone marrow transplant is the only curative means of treatment for thalassemia major patients. If performed early, usually below 5 years of age, in an adequately chelated child, the cure rate approaches 90% [8]. The first bone marrow transplant was done in 2012 in Nepal, and since then, more than 150 transplants have been done at the Civil Service Hospital at a very affordable price compared to the rest of the world [9, 10]. The average cost of bone marrow transplant for thalassemia at Civil Service Hospital patients is 11,500 USD (according to personal communication with the President of Nepal Thalassemia Society). Studies have shown that bone marrow transplant is a cost‐effective treatment modality in low‐middle‐income countries (LMICs). The published data from LMICs suggest that bone marrow transplantation improves the quality of life compared to lifelong blood transfusion and iron chelation in thalassemia major patients [11]. Based on our findings of the annual cost of 70,000–250,000 NRS (USD 533–1906) for conventional treatment, with such a high‐rate complication, the concerned health stakeholders, along with hematologists, should develop a proper advocacy plan to convince the government to make bone marrow transplants free of costs like a renal transplant for chronic kidney disease to decrease the health‐care burden and quality of life of thalassemia patients.

The statement “Prevention is better than cure” holds very true in a condition like β‐thalassemia major, which poses a significant economic burden on health services and the families of affected children. Several countries have established an effective way of screening and have made tremendous progress [12]. Although screening and testing are culturally accepted, lack of awareness and advocacy, shortage of dedicated funds, and diagnostic and interpretative challenges have made screening almost nonexistent in Nepal.

Nepal Thalassemia Society has played a pivotal role in maintaining the registry of thalassemia patients. It is a nonprofit, nongovernment patient organization and was established to work on thalassemia‐related advocacy programs. Since its establishment, it has provided a space for blood transfusion under the supervision of trained medical personnel. Although blood transfusion is considered a lifeline of thalassemia care, patients need multidisciplinary medical care to live a normal life. With the start of clinical hematology training in the country, the government and concerned stakeholders must work together to initiate thalassemia care from an academic institution.

In conclusion, thalassemia patients in Nepal deserve better care, and there is a dire need for short‐ and long‐term plans to combat thalassemia. Currently, the best short‐term goal would be to establish a heavily subsidized bone marrow transplant program in an experienced academic institution. In the long run, the government should invest more in improving the blood supply through national banking systems, launching effective screening and prenatal diagnosis campaigns, and developing a solution to tackle frequent shortages of iron‐chelating agents.

## AUTHOR CONTRIBUTIONS

Bishesh Sharma Poudyal designed the research and wrote the manuscript. Anirudra Devkota  collected and analyzed the data. Peter Kouides revised the paper. All authors have read and approved the final version of the manuscript.

## CONFLICT OF INTEREST STATEMENT

The authors declare no conflicts of interest.

## FUNDING INFORMATION

None.

## ETHICS STATEMENT

The study has been approved from Ethical Board Review of Nepal Thalassemia Society.

## Data Availability

The data that support the findings of this study are available on request from the corresponding author. The data are not publicly available due to privacy or ethical restrictions.
